# A Preliminary Study on the Qualitative and Quantitative Changes of Amino Acids in Whole Apple, Apple Peel, and Flesh Samples Grown in Lithuania

**DOI:** 10.3390/plants14091330

**Published:** 2025-04-28

**Authors:** Aurita Bračiulienė, Vaidotas Žvikas, Mindaugas Liaudanskas, Valdimaras Janulis

**Affiliations:** 1 Department of Pharmacognosy, Faculty of Pharmacy, Lithuanian University of Health Sciences, Sukilėlių av. 13, LT-50162 Kaunas, Lithuania; mindaugas.liaudanskas@lsmu.lt (M.L.); valdimaras.janulis@lsmu.lt (V.J.); 2 Institute of Pharmaceutical Technologies, Faculty of Pharmacy, Lithuanian University of Health Sciences, Sukilėlių av. 13, LT-50162 Kaunas, Lithuania; vaidotas.zvikas@lsmu.lt

**Keywords:** apple, apple peel, apple flesh, amino acids

## Abstract

Amino acids are vital gradient compounds involved in protein synthesis and the regulation of physiological functions. Ten essential amino acids cannot be produced endogenously and must be obtained through dietary sources of animal or plant origin. Apples are among the most widely consumed fruits globally and contain not only vital nutrients such as carbohydrates, fatty acids, organic acids, and amino acids but also a rich variety of bioactive compounds, including flavonoids and phenolic and triterpenic acids. Due to their diverse range of health-promoting compounds, apples could serve as a potential plant-based source of amino acids. Scientific literature provides fragmented data describing the qualitative and quantitative variation of amino acid composition in apples and their different parts. The purpose of this study was to determine the variation in the qualitative and quantitative composition of amino acids in whole apples, as well as in their peel and flesh samples, grown under Lithuanian climatic conditions. This study investigated 10 different apple cultivars, grown in Lithuania. A total of 15 free amino acids were identified using the UHPLC–MS/MS methodology, including 7 essential and 8 nonessential amino acids. In the apple sample, the predominant amino acid was aspartic acid (Asp). The highest content of Asp was found in apple flesh (742.73 ± 37.14 μg/g dw, *p* < 0.05), followed by whole apple (705.32 ± 35.27 μg/g dw, *p* < 0.05), and apple peel (370.78 ± 18.54 μg/g dw, *p* < 0.05) samples of the ‘Lodel’ cultivar. The distribution of total amino acid content (TAAC) in different apple parts is presented in descending order: apple flesh > whole apple > apple peel. The Lithuanian apple cultivars—‘Alva’ (547.26 ± 27.36 μg/g dw–998.13 ± 49.91 μg/g dw), ‘Lodel’ (561.85 ± 28.09 μg/g dw–954.24 ± 47.62 μg/g dw), and ‘Rubin’ (132.92 ± 6.65 μg/g dw–835.08 ± 41.75 μg/g dw)—were identified as those that accumulated the highest TAAC in their fruit samples.

## 1. Introduction

Amino acids are defined as essential gradient compounds with a significant role in tissue protein synthesis. Due to variations in their side chains, amino acids exhibit distinct biochemical properties and functions. In plants, amino acids serve as precursors to volatile compounds, proteins, nucleic acids, and other organic molecules. Moreover, certain amino acids also act as initial substrates for the biosynthesis of phenolic compounds, an important group of secondary metabolites involved in plant adaptation to biotic and abiotic stresses [1,2]. Furthermore, different amino acids in plants perform various physiological roles and fulfill essential functions, including acting as osmolytes, altering enzyme activity, modulating stomatal opening, regulating seed germination, facilitating ion transport, and detoxifying heavy metals [2,3]. The nutrient requirements of amino acids depend on species, developmental stage, physiological status, the microbiota in the lumen of the small intestine, environmental factors, and pathological states [4,5,6]. Scientific results have described the positive outcomes of amino acids in the treatment of diseases such as infertility, intestinal disorders, neurological dysfunction, preventing cardiovascular disease, protection from arteriosclerosis, and diabetes mellitus [7,8,9]. The growing consumer interest in functional foods has encouraged researchers to study the phytochemical composition of dietary foods, such as fruits and vegetables, in more detail.

Apples (*Malus domestica* Borkh.) are among the most economically important and widely consumed fruits in the world, with 95.8 million tons produced annually in 2022 [10]. It is widely consumed fresh or as apple products in processed forms such as juices, jam, cider, vinegar, and dried fruit [1,11,12,13]. The widespread use of apples and their processed products is determined not only by the combination of organoleptic properties but also by their nutritional value and applicability for health purposes. Phytochemical composition studies have revealed that apples are important natural sources of vitamins, sugars, dietary fiber, minerals, organic acids, fatty acids, amino acids, triterpenic acids, and phenolic compounds [14,15,16,17,18,19]. Phenolic compounds are a relevant group of secondary metabolites found in apples, which are extensively studied due to their diverse biological effects and practical applications [20,21,22]. However, the literature still provides limited information about the variation in the qualitative and quantitative composition of amino acids in apples and their individual parts, which are significant components for human health. Amino acids are broadly classified as nutritionally essential or nonessential [6]. Essential amino acids (leucine, isoleucine, methionine, phenylalanine, arginine, histidine, tryptophan, valine, threonine, and lysine) have carbon skeletons that are synthesized by plants, while nonessential amino acids (alanine, β-alanine, asparagine, cysteine, glutamic acid, aspartic acid, glycine, proline, serine, and tyrosine) are synthesized by both plants and humans [23]. Essential amino acids must be provided through animal or plant-based foods to sustain human health [6,23,24].

The quality of apples depends on various biotic and abiotic factors. To ensure apple quality and nutritional value, it is crucial to consider the genetic origin of the fruit trees, the management of fruit growth physiology, and environmental factors. The genotype, changes in gene expression during fruit development and growth, geographical location, environmental climatic conditions, soil geochemical composition, light radiation, and agro-technological processes are among the most significant factors determining the mineral and phytochemical composition of apples [25,26,27,28]. Fragmentary research data are presented on changes in the amino acid profile in different parts of apples. Most scientific studies have focused on evaluating variations in amino acids in apple juice or cider [29,30,31]. It is important to determine the patterns of variation in the amino acid profile of apples and to identify cultivars that accumulate the highest amounts of amino acids in their fruits. More detailed knowledge of the variability in the composition of apples from different cultivars will be helpful in the future selection of apple genotypes with improved nutritional quality and suitable processing characteristics.

In the daily diet, consumed apples can serve as an alternative source of naturally derived amino acids. Therefore, it is important to study the qualitative and quantitative composition of amino acids to identify apple cultivars that accumulate the highest amounts of these compounds and to assess the changes in the amino acid profile in different parts of the apples, such as the peel and flesh. These studies are important in the search for promising plant raw materials and biological markers that could be used to determine the cultivar of fruits. Moreover, these research findings are highly important in the food industry for the fermentation process in apple-based products and for the development of new innovative dietary supplements and cosmetic products. The scientific literature does not provide data describing changes in the qualitative and quantitative composition of amino acids in different apple cultivars grown under Lithuanian climatic conditions. The aim of the study was to determine the variation in the qualitative and quantitative composition of amino acids in whole apples, as well as in their peel and flesh samples, grown under Lithuanian climatic conditions.

## 2. Results

### 2.1. Variation in Amino Acids Content in Whole Apple, Apple Peel, and Flesh Samples

Using the UHPLC–MS/MS methodology, 15 amino acids (7 essential and 8 nonessential amino acids) were identified and quantitatively determined in whole apple, apple peel, and flesh samples.

**Figure 1:**
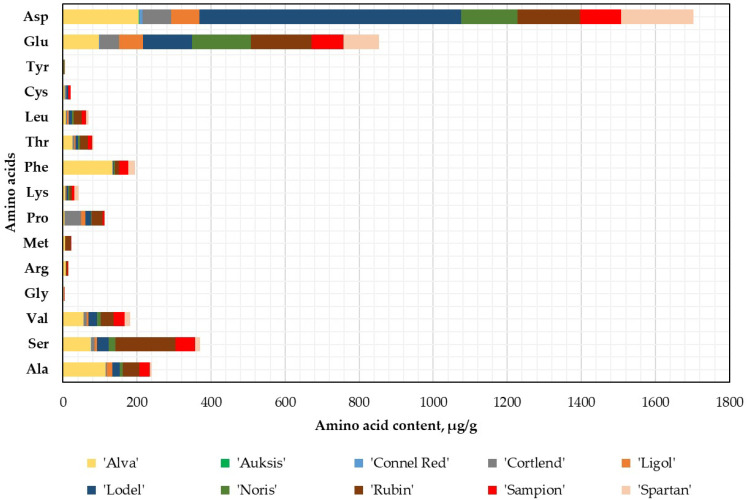
Amino acid content in the whole apple samples.

After conducting the qualitative and quantitative analysis of free amino acids, it was determined that aspartic acid (Asp) was the predominant amino acid in whole apple samples, except for the sample of the ‘Noris’ cultivar, where glutamic acid (Glu) was predominant. The statistically significant highest content of Asp (705.32 ± 35.27 μg/g dw, *p* < 0.05) was found in the whole apple samples of the ‘Lodel’ cultivar, while the lowest (1.0 ± 0.05 μg/g dw) was observed in the apple samples of the ‘Auksis’ cultivar (Figure 1).

Glu was the second most abundant amino acid in the whole apple samples, with its levels being 1 to 6 times lower than those of Asp. The highest content of Glu (164.61 ± 8.23 μg/g dw) was determined in the whole apple samples of the ‘Rubin’ cultivar, and its levels did not differ statistically significantly from those determined in the apple samples of the ‘Noris’ cultivar (Figure 1). Among the 15 identified amino acids, the highest levels of seven were found in the apple samples of the ‘Alva’ cultivar: Ala (114.87 ± 5.74 μg/g dw, *p* < 0.05), Val (56.31 ± 2.82 μg/g dw, *p* < 0.05), Gly (1.88 ± 0.09 μg/g dw, *p* < 0.05), Arg (7.35 ± 0.37 μg/g dw, *p* < 0.05), Phe (133.35 ± 6.67 μg/g dw, *p* < 0.05), Thr (26.19 ± 1.31 μg/g dw, *p* < 0.05), and Cys (5.14 ± 0.26 μg/g dw, *p* > 0.05) (Figure 1). It was evaluated that, among the identified amino acids, the lowest content 0.03 ± 0.001 μg/g dw and 0.04 ± 0.002 μg/g dw were found for Tyr in the apple samples of the ‘Auksis’ and ‘Connel Red’ cultivars, respectively (Figure 1). The ‘Auksis’ cultivar apple accumulated the lowest levels of free amino acids. It was determined that out of the 15 identified free amino acids, only 4 (Ser, Leu, Tyr, and Asp) were quantified in the whole apple samples of the ‘Auksis’ cultivar (Figure 1).

**Figure 2:**
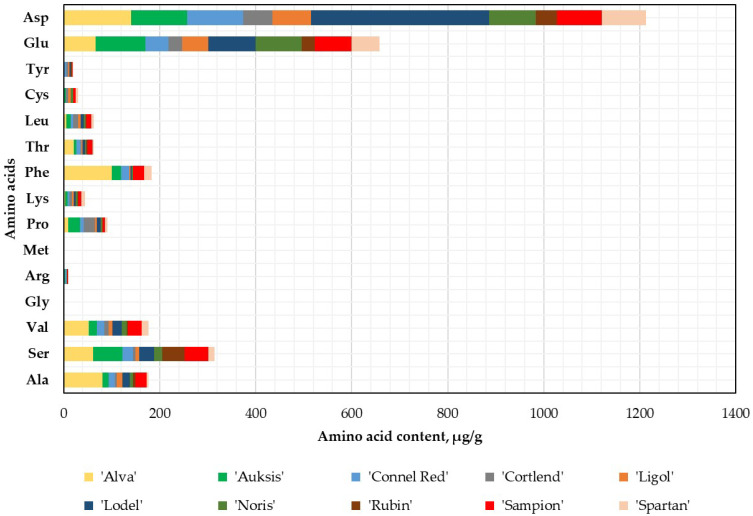
Amino acid content in the apple peel samples.

The qualitative and quantitative variations in the amino acid composition of the apple peel samples were analyzed. Asp and Glu remained the predominant amino acids in the apple peel samples (Figure 2). The statistically significant highest Asp content (370.78 ± 18.54 μg/g dw, *p* < 0.05) was found in the apple peel samples of the ‘Lodel’ cultivar, which was half the amount found in the whole apple samples, while the highest Glu content (104.24 ± 27.25 μg/g dw) was found in the apple peel samples of ‘Auksis’ cultivar.

During the analysis, it was determined that highest levels of amino acids Ala (81.56 ± 4.08 μg/g dw, *p* < 0.05), Ser (61.25 ± 3.06 μg/g dw, *p* > 0.05), Val (52.47 ± 2.62 μg/g dw, *p* < 0.05), Gly (0.47 ± 0.02 μg/g dw, *p* < 0.05), Arg (2.46 ± 0.12 μg/g dw, *p* > 0.05), Phe (99.84 ± 4.99 μg/g dw, *p* < 0.05), and Thr (20.96 ± 1.05 μg/g dw, *p* < 0.05) were found in the apple peel samples of the ‘Alva’ cultivar. The lowest amino acid content of 0.02 ± 0.001 μg/g dw was determined for Arg in the apple peel samples of the ‘Ligol’ cultivar (Figure 2). In the apple peel samples of all tested cultivars, Met was not identified or quantified. Gly was only determined in the apple peel samples of the ‘Alva’ cultivar (Figure 2).

**Figure 3:**
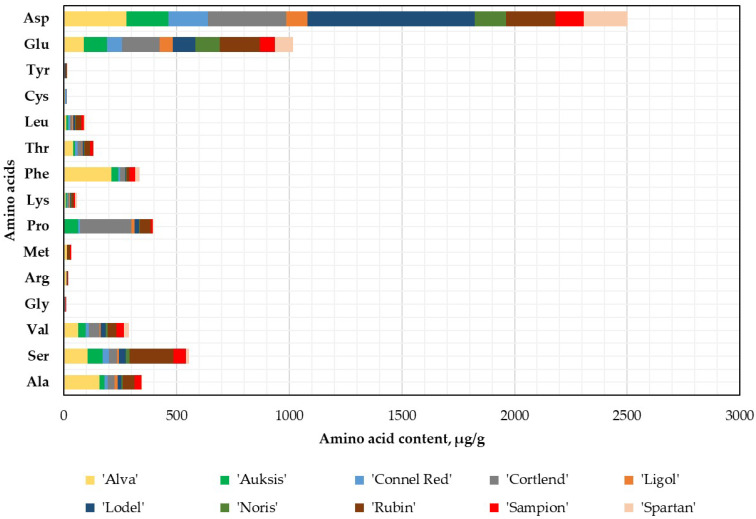
Amino acid content in the apple flesh samples.

This study revealed that Asp and Glu dominated in the apple flesh samples of different cultivars. The statistically highest Asp content (742.73 ± 37.14 μg/g dw, *p* < 0.05) was found in the apple flesh samples of the ‘Lodel’ cultivar. Meanwhile, the lowest amount (94.08 ± 4.70 μg/g dw) was determined in the apple flesh samples of the ‘Ligol’ cultivar (Figure 3).

The highest Glu content (178.48 ± 8.92 μg/g dw) was observed in the apple flesh samples of the ‘Rubin’ cultivar, as well as in the whole apple samples. In the apple flesh samples of the ‘Alva’ cultivar, the highest levels of five amino acids were determined: Ala (157.12 ± 7.86 μg/g dw, *p* < 0.05), Val (63.60 ± 3.18 μg/g dw, *p* < 0.05), Arg (11.01 ± 0.55 μg/g dw, *p* < 0.05), Phe (211.91 ± 10.60 μg/g dw, *p* < 0.05), and Thr (40.56 ± 2.03 μg/g dw, *p* < 0.05), which were 1.2–4.5 times higher than the levels found in the apple peel samples (Figure 3).

During the study, Asp remained the predominant amino acid in whole apples, apple peel, and flesh samples from all tested apple cultivars. Depending on the cultivar and apple part, the levels of Glu, Phe, Pro, and Ser varied, with these free amino acids consistently being among the most commonly detected in the apple samples.

The quantitative analysis revealed that the total amino acid content (TAAC) in the whole apple samples ranged from 1.18 ± 0.06 μg/g dw to 954.24 ± 47.71 μg/g dw. The statistically significant highest content (954.24 ± 47.71 μg/g dw, *p* < 0.05) was found in the whole apple samples of the ‘Lodel’ cultivar, while the lowest content (1.18 ± 0.06 μg/g dw, *p* < 0.05) was observed in the whole apple samples of the ‘Auksis’ cultivar. During the analysis of apple peel samples, the highest TAAC (561.85 ± 18.54 μg/g dw) was found in the peel of the ‘Lodel’ cultivar, which was approximately half of the content found in whole apple samples. Meanwhile, the lowest TAAC (132.93 ± 6.65 μg/g dw) was determined in the apple peel samples of the ‘Rubin’ cultivar. The highest total amino acid levels were found in the apple flesh samples of tested cultivars. The highest TAAC (998.13 ± 49.91 μg/g dw) was observed in the apple flesh samples of the ‘Alva’ cultivar, while the lowest (201.23 ± 10.06 μg/g dw) was found in the apple flesh samples of the ‘Ligol’ cultivar.

The qualitative and quantitative analysis of free amino acids showed that the highest levels of amino acids are accumulated in the apple flesh. The distribution of total amino acid content (TAAC) in different apple parts across all cultivars is presented in descending order: apple flesh (201.23–998.13 μg/g dw) > whole apple (1.18–954.24 μg/g dw) > apple peel (132.93–561.85 μg/g dw). The TAAC in the apple flesh samples of different apple cultivars was 1–6 times higher than in the apple peel samples and 1–4 times higher than in the whole apple samples.

**Figure 4:**
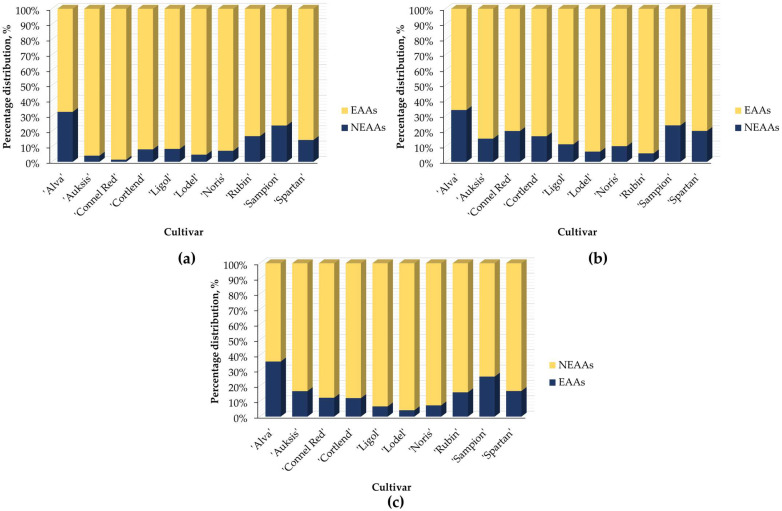
The percentage distribution of essential (EAAs) and nonessential amino acids (NEAAs) in different apple cultivars: (**a**) percentage distribution of EAAs and NEAAs in the whole apple samples; (**b**) percentage distribution of EAAs and NEAAs in the apple peel samples; (**c**) percentage distribution of EAAs and NEAAs in the apple flesh samples. Abbreviations: EAAs—essential amino acids; NEAAs—nonessential amino acids.

The percentage distribution of essential (EAAs) and nonessential (NEAAs) amino acids in the apple samples and their individual parts was evaluated in relation to the apple cultivar (Figure 4).

The content of EAAs were found in the whole apple samples, listed in descending order by apple cultivar: ‘Alva’ (32.65%) > ‘Sampion’ (23.72%) > ‘Rubin’ (16.77%) > ‘Spartan’ (14.26%) > ‘Ligol’ (8.43%) > ‘Cortlend’ (8.13%) > ‘Noris’ (7.20%) > ‘Lodel’ (4.67%) > ‘Auksis’ (4.00%) > ‘Connel Red’ (1.40%) (Figure 4a). The levels of EAAs were determined in the apple peel samples, arranged by cultivar in diminishing order: ‘Alva’ (33.85%) > ‘Sampion’ (23.80%) > ‘Spartan’ (20.20%) > ‘Connel Red’ (20.12%) > ‘Cortlend’ (16.70%) > ‘Auksis’ (15.14%) > ‘Ligol’ (11.45%) > ‘Noris’ (10.21%) > ‘Lodel’ (6.67%) > ‘Rubin’ (5.52%) (Figure 4b). The amounts of EAAs were observed in the apple flesh samples, arranged by cultivar in descending order: ‘Alva’ (35.90%) > ‘Sampion’ (26.06%) > ‘Spartan’ (16.66%) > ‘Auksis’ (16.55%) > ‘Rubin’ (15.79%) > ‘Connel Red’ (12.27%) > ‘Cortlend’ (12.03%) > ‘Noris’ (7.31%) > ‘Ligol’ (6.64%) > ‘Lodel’ (4.14%) (Figure 4c).

The highest content of essential amino acids (EAAs) was found in the apple samples of the ‘Alva’ cultivar. Additionally, apples from the ‘Sampion’ and ‘Spartan’ cultivars were identified as potential sources of EAAs.

**Figure 5:**
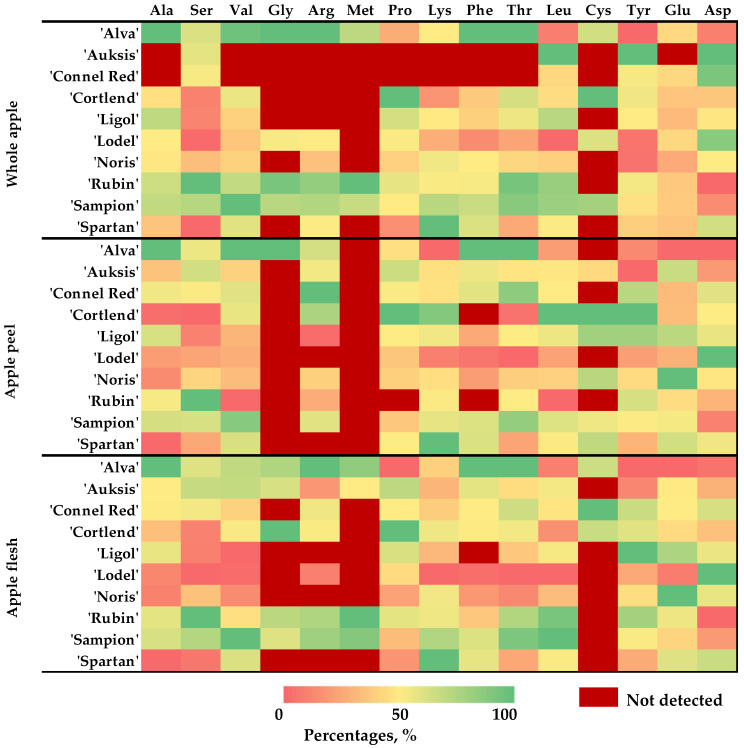
Heatmap revealing the percentage distribution of amino acids in the whole apple, apple peel, and flesh samples (normalized quantity, percentage).

The percentage distribution of free amino acids in the samples of different apple cultivars was evaluated (Figure 5). In the whole apple samples of the ‘Auksis’, ‘Connel Red’, and ‘Lodel’ cultivars, Asp dominated, constituting 84.76%, 77.99%, and 73.91% of the TAAC, respectively. In the whole apple samples of the remaining apple cultivars, Asp acid constituted between 24.61% and 54.03% of the TAAC. In the whole apple samples of the ‘Noris’ cultivar, Glu predominated, constituting 43.19% of the TAAC. In the whole apple samples of the ‘Alva’ cultivar, Phe was the second most predominant amino acid after Asp, constituting 17.84% of the TAAC. In the whole apple samples of the ‘Cortland’ cultivar, Pro was the third most abundant amino acid, constituting 21.22% of the TAAC. In the whole apple samples of the ‘Rubin’ cultivar, three amino acids were predominant in similar amounts: Ser (23.43%), Glu (23.92%), and Asp (24.61%).

In the apple peel samples, Asp constituted from 25.70% to 65.99% of the TAAC. In the apple peel samples of the ‘Lodel’ cultivar, Asp and Glu predominated, constituting 65.99% and 17.54% of the TAAC, accordingly. In the apple peel samples of the ‘Alva’ cultivar, Phe remained the second most abundant amino acid, constituting 18.24% of the TAAC. In the apple peel samples of the ‘Cortland’ cultivar, Pro remained the third most abundant amino acid, constituting 14.98% of the TAAC. In the apple peel samples of the ‘Rubin’ cultivar, the highest amount was found in Ser, which constituted the largest portion, 35.09%, of the TAAC.

The percentage distribution of Asp in the apple flesh samples varied from 26.54% to 77.98%. In the apple flesh samples of the ‘Lodel’ cultivar, as well as in the whole apple and apple peel samples, Asp constituted the largest portion, 77.98% of the TAAC. The lowest distribution of Asp, 26.54%, was found in the apple flesh samples of the ‘Rubin’ cultivar. In the apple flesh samples of the ‘Noris’ cultivar, Glu constituted 36.38% of the TAAC and, like in the whole apple and apple peel samples, remained the second most abundant amino acid. In the apple flesh samples of the ‘Alva’ cultivar, Phe constituted 21.23% of the TAAC and remained the second most abundant amino acid. This trend of Phe variation was also observed in the whole apple and apple peel samples. In the apple flesh samples of the ‘Cortland’ cultivar, Pro constituted 24.28% of the TAAC and remained the second most predominant amino acid. In the apple flesh samples of the ‘Rubin’ cultivar, Ser constituted 23.20% of the TAAC and was the second most abundant amino acid. This trend of change was also established in the whole apple and apple peel samples, where Ser remained one of the three predominant amino acids.

The qualitative and quantitative analysis showed that the amino acid content in the apple samples varied depending on the cultivar. The highest amino acid content was observed in the fruit samples of the ‘Lodel’, ‘Alva’, and ‘Rubin’ cultivars. The research results revealed that in the apple samples of the ‘Alva’ cultivar, unlike in the fruit samples of other tested apple cultivars, one of the most frequently detected amino acids, after Asp and Glu, was the essential amino acid Phe. Meanwhile, in the apple samples of the ‘Cortland’ cultivar, it was Pro, and, in the samples of the ‘Rubin’ cultivar, it was Ser. The highest levels of essential amino acids were detected in the apple samples of the ‘Alva’, ‘Sampion’, and ‘Spartan’ cultivars.

**Figure 6:**
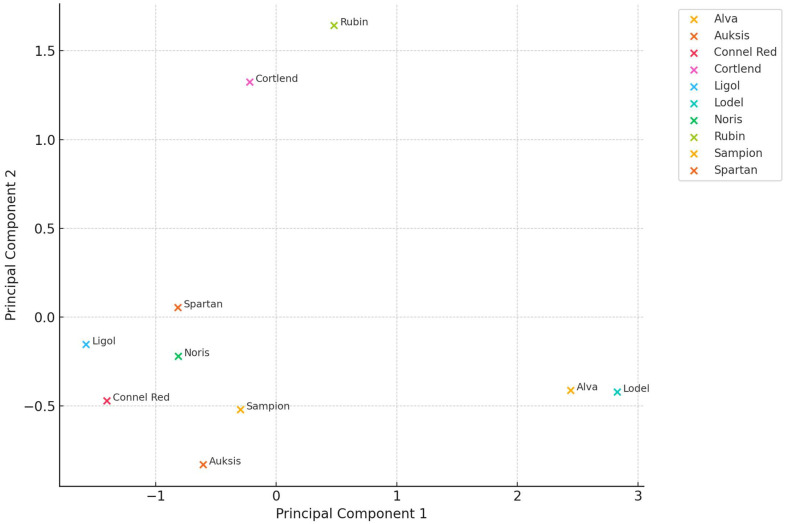
PCA of total amino acid content in different apple parts.

To elucidate the variability in free amino acid composition across different apple cultivars and fruit tissues, a principal component analysis (PCA) was conducted using the total amino acid content (μg/g dw) of whole apple, peel, and flesh samples. The first two principal components (PC1 and PC2) accounted for 88.47% of the total variance—68.31% by PC1 and 20.16% by PC2—indicating a high level of explanatory power and justifying the dimensionality reduction (Figure 6). The PCA biplot demonstrated distinct clustering patterns among cultivars, reflecting their biochemical differentiation.

Cultivars such as ‘Lodel’ and ‘Alva’ were positioned on the right side of the PCA plot, suggesting a high accumulation of total amino acids, particularly in the flesh tissue. Conversely, cultivars such as ‘Auksis’, ‘Sampion’, and ‘Spartan’ grouped toward the left or lower quadrants, indicating lower overall amino acid content. ‘Rubin’ and ‘Cortlend’ were differentiated along the second component axis, likely due to elevated amino acid levels in both peel and flesh.

These findings highlight the discriminative capacity of PCA in revealing cultivar-specific amino acid profiles and support its application in nutritional phenotyping, varietal classification, and targeted breeding strategies aimed at enhancing the nutritional quality of apples.

### 2.2. Variation in Correlation Coefficient Between Different Amino Acids in Whole Apple, Apple Peel, and Flesh Samples

**Figure 7:**
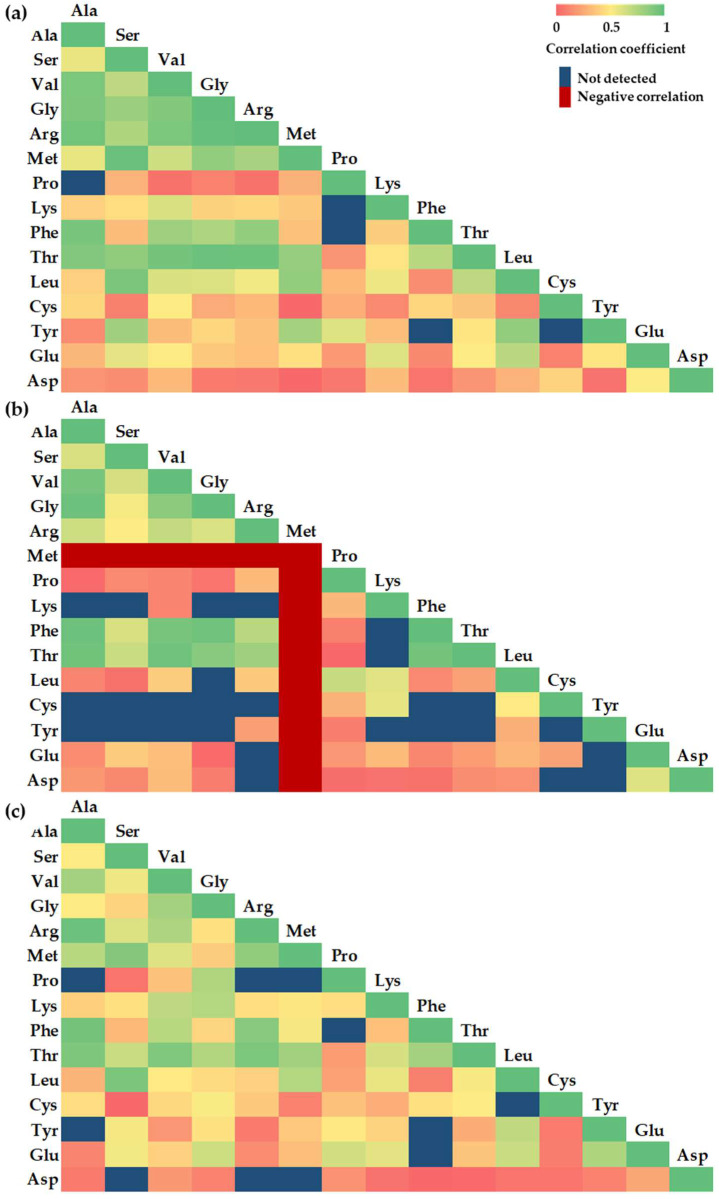
Correlation matrix based on Pearson’s correlation coefficient between different amino acids: (**a**) heatmap reveals the variation in the correlation coefficient of amino acids in the whole apple samples; (**b**) heatmap reveals the variation in the correlation coefficient of amino acids in the apple peel samples; (**c**) heatmap reveals the variation in the correlation coefficient of amino acids in the apple flesh samples. Abbreviations: Ala—L-alanine; Ser—L-serine; Val—L-valine; L-Gly—L-glycine; Arg—L-arginine; Met—L-methionine; Pro—L-proline; Lys—L-lysine; Phe –L-phenylalanine; Thr—L-threonine; Leu—L-leucine; Cys—L-cystine; Tyr—L-tyrosine; Glu—L-glutamic acid; Asp—L-aspartic acid.

The strength of the correlation between the contents of free amino acids was estimated. The matrix created allows for a comprehensive comparison of the correlation relationships between the characteristics selected for evaluation. The intensity of colors is directly proportional to the correlation coefficient (Figure 7).

Based on the results of the analysis of whole apple samples, statistically significant very strong positive correlations (the correlation coefficient ranged from r = 0.911 to r = 0.992, *p* < 0.05) were observed in 12 out of 105 cases (Figure 7a). The strongest correlation, r = 0.992, *p* < 0.05, was found between the quantities of Arg and Gly in the whole apple sample. Statistically significant strong correlations (the correlation coefficient ranged from r = 0.737 to r = 0.906, *p* < 0.05) were found in 18 out of 105 cases. Statistically significant moderate correlations (the correlation coefficient ranged from r = 0.419 to r = 0.690, *p* < 0.05) were found in 29 out of 105 cases. Evaluation of the correlation strength between amino acid content in the apple peel samples revealed very strong correlations (correlation coefficient ranged from r = 0.933 to r = 0.965, *p* < 0.05) in 8 out of 105 cases (Figure 7b). Statistically significant strong correlations (the correlation coefficient ranged from r = 0.787 to r = 0.873, *p* < 0.05) were found in 3 out of 105 cases. Statistically significant moderate correlations (the correlation coefficient ranged from r = 0.444 to r = 0.692, *p* < 0.05) were found in 17 out of 105 cases. Based on the results of the analysis of the apple flesh samples, statistically significant very strong positive correlations (the correlation coefficient ranged from r = 0.916 to r = 0.972, *p* < 0.05) were observed in 6 out of 105 cases (Figure 7c). Statistically significant strong correlations (the correlation coefficient ranged from r = 0.710 to r = 0.905, *p* < 0.05) were found in 20 out of 105 cases. Statistically significant moderate correlations (the correlation coefficient ranged from r = 0.412 to r = 0.703, *p* < 0.05) were found in 36 out of 105 cases.

A total of 315 correlation coefficients were verified and compared. All the established very strong, strong, and moderate correlations were statistically significant. The highest number of very strong correlations between amino acid content was observed in the whole apple samples, while strong and moderate correlations were found in the apple flesh samples.

## 3. Discussion

Amino acids play a fundamental role in cellular physiology, acting as building blocks for proteins and contributing to numerous biochemical and regulatory processes. They are essential for tissue development, differentiation, energy metabolism, and immune function [32]. Although over 300 amino acids have been identified, only 20 participate in ribosomal protein synthesis, of which 9 are classified as essential, because they must be obtained through dietary intake [33]. Fruits and vegetables, including apples, are important sources of these compounds and are increasingly investigated as potential functional foods.

Apples (*Malus domestica* Borkh.) are widely consumed and rich in nutrients and bioactive compounds with antioxidant, anti-inflammatory, antimicrobial, and metabolic health-promoting effects [34,35,36,37,38,39,40,41,42,43,44]. Despite considerable focus on phenolic compounds in apples, the variability in amino acid profiles remains less understood, particularly in relation to specific cultivars and plant tissues. Using UHPLC–MS/MS, our study identified 15 free amino acids (7 essential and 8 nonessential) in whole apples, as well as in peel and flesh samples from 10 cultivars grown under Lithuanian climatic conditions. This expands the knowledge of apple biochemical composition and its potential health relevance.

One of the key findings was the predominance of aspartic acid (Asp) across all tissues and most cultivars, particularly in ‘Lodel’, where it constituted up to 77.98% of the total amino acid content (TAAC) in the flesh. This result corroborates previous studies conducted in Türkiye [12], China [24,45], and Brazil [13]. Asp is a key node in amino acid biosynthesis pathways, linking the tricarboxylic acid (TCA) cycle with nitrogen metabolism, and serves as a precursor for essential amino acids such as lysine, threonine, methionine, and isoleucine [46]. It also contributes to cellular energy homeostasis and mitochondrial function [46,47].

Recent studies have revealed the role of Asp in plant defense and stress response. For example, Wu et al. (2022) demonstrated that exogenous Asp application improves salt tolerance by enhancing antioxidant enzyme activities and osmotic adjustment [48]. These findings suggest that elevated Asp in apples may serve as a physiological adaptation to environmental stress, especially relevant under the fluctuating climatic conditions of Lithuania. Besides Asp, other amino acids such as glutamic acid (Glu), phenylalanine (Phe), proline (Pro), and serine (Ser) were found in significant concentrations. Glu, the second most abundant amino acid in most cultivars (especially ‘Rubin’ and ‘Noris’), is a central compound in nitrogen assimilation, transamination reactions, and the biosynthesis of γ-aminobutyric acid (GABA), a stress-related metabolite [47]. Phe, notably abundant in the ‘Alva’ cultivar, serves as a precursor to phenylpropanoids, compounds essential for plant structural integrity and defense against pathogens and UV radiation [14,22]. Elevated Phe levels may, thus, reflect a cultivar’s capacity to synthesize protective secondary metabolites. Proline, which accumulated prominently in the ‘Cortland’ cultivar, is well-known for its role in osmoprotection and redox balance. Its accumulation is a common adaptive response to drought, salinity, and cold stress [2,3]. This supports the notion that Pro content may indicate stress resilience potential among cultivars. Serine, found in high proportions in ‘Rubin’, is involved in one-carbon metabolism and contributes to the biosynthesis of amino acids, phospholipids, and other biomolecules. Its role in stress signaling and metabolic regulation further enhances its biological relevance.

Variation in amino acid content between cultivars and apple parts highlights the influence of genetic and environmental factors on biosynthetic pathways. Cultivars such as ‘Alva’, ‘Lodel’, and ‘Rubin’ demonstrated superior profiles in both total and essential amino acids, marking them as valuable candidates for breeding programs focused on functional food development and climate-resilient crops.

Multivariate analyses, including principal component analysis (PCA), confirmed that amino acid profiles can serve as discriminative markers for cultivar classification and nutritional phenotyping. Additionally, correlation matrix analysis revealed strong interdependencies among several amino acids, suggesting coordinated regulation or common metabolic origins.

The broader physiological significance of amino acids extends beyond plant metabolism. In human health, essential amino acids (e.g., Val, Ile, Leu) are crucial for muscle repair, while nonessential amino acids (e.g., Arg, Gly, Gln, Pro) regulate gene expression, immune responses, antioxidant systems, and metabolic homeostasis [5,49,50,51,52,53,54,55,56,57,58,59,60,61,62,63,64,65]. Aspartic acid, in particular, is involved in cell proliferation, mitochondrial function, and leucocyte metabolism [46,47]. Alanine is a primary substrate for hepatic gluconeogenesis and is critical for immune cell function [47].

In conclusion, the dominance of Asp and substantial levels of Glu, Phe, Pro, and Ser in Lithuanian apple cultivars reflect not only their nutritional significance but also their contribution to plant stress tolerance. These results emphasize the importance of comprehensive amino acid profiling in apples, which may support targeted breeding strategies, promote nutritional enhancement, and expand the use of apples in functional food formulations.

## 4. Materials and Methods

### 4.1. Fruit Materials

This study investigated 10 different apple cultivars: ‘Alva’, ‘Auksis’, ‘Connell Red’, ‘Cortland’, ‘Ligol’, ‘Lodel’, ‘Noris’, ‘Rubin’, ‘Sampion’, and ‘Spartan’. Apple samples were prepared at the Institute of Horticulture (Babtai, Lithuania), a branch of the Lithuanian Research Center for Agriculture and Forestry (coordinates: 55.600° N, 23.480° E).

### 4.2. Chemicals and Solvents

All solvents, reagents, and standards used were of analytical grade. Solvents used were purified water (Millipore^®^, Bedford, MA, USA), 99.9% acetonitrile, 99.9% formic acid, and ammonium formate (Sigma-Aldrich^®^, Steinheim, Germany). Standards of amino acids used were L-alanine (Ala), L-serine (Ser), L-valine (Val), L-glycine (Gly), L-arginine (Arg), L-methionine (Met), L-proline (Pro), L-lysine (Lys), L-phenylalanine (Phe), L-threonine (Thr), L-leucine (Leu), L-cystine (Cys), L-tyrosine (Tyr), L-glutamic acid (Glu), and L-aspartic acid (Asp) (Sigma-Aldrich^®^, Steinheim, Germany).

### 4.3. Preparation of Whole Apple, Apple Peel, and Flesh Extracts

The lyophilized samples of whole apples, apple peels, and flesh were prepared as described by Butkeviciute et al. [66]. During the analysis, 1.0 g (exact weight) of lyophilized apple powder was weighed, added to 10 mL of water, and extracted in an ultrasonic bath (Elmasonic P, Elma Schmidbauer GmbH, Singen, Germany) for 15 minutes at 22 ± 1 °C, at a frequency of 80 kHz and power of 1017 W. Afterward, the prepared apple extracts were filtered through a membrane filter with a pore size of 0.20 µm into a 10 mL volumetric flask. The extraction of the lyophilized apple samples was repeated three times. The prepared whole apple, apple peel, and flesh extracts were stored in dark glass containers.

### 4.4. Assay of Amino Acids by UPLC-MS/MS

Amino acid analysis in plant samples was performed using an Acquity H-Class UPLC system (Waters, Milford, MA, USA) coupled with a Xevo TQD mass spectrometer (Waters, Milford, MA, USA). One microliter of extract was injected into a BEH Amide column (150 mm × 2.1 mm, 1.7 µm; Waters, Milford, MA, USA), maintained at a temperature of 25 °C. The mobile phase consisted of an aqueous solution of 10 mmol ammonium formate with 0.125% formic acid (eluent A) and acetonitrile (eluent B), delivered at a flow rate of 0.6 mL/min. A gradient elution method was applied with the following settings: 0–1 min, 95% B; 1–3.9 min, 70% B; 3.9–5.1 min, 30% B; at 5.1–6.4 min, the column was flushed with 70% eluent A; at 6.5 min, the gradient was returned to the initial composition, with a total run time of 10 minutes. Mass spectrometer parameters were set as follows: positive electrospray ionization at +3.5 kV, cone voltage at 30 V, desolvation gas flow at 800 L/h, desolvation temperature at 400 °C, and ion source temperature at 120 °C. Amino acid identification in the extracts was performed by comparing their retention times and MS/MS spectra with those of analytical grade standards. Quantitative analysis was carried out using the standard dilution method, and linear regression models were applied for the quantification of individual amino acids.

### 4.5. Statistical Analysis

The analysis of the UHPLC–MS/MS data was performed using Microsoft Office Excel 2015 (Microsoft, Redmond, WA, USA) and SPSS Statistics 25 (SPSS Inc., Chicago, IL, USA) computer programs. Experiments were repeated in triplicate, with results presented as arithmetic mean ± standard deviation (SD) of replicates. Univariate analysis of variance (ANOVA) was applied to establish whether the differences between the compared data were statistically significant. The hypothesis about the equality of variances was verified by applying Levine’s test. If the variances of independent variables were found to be equal, Tukey’s post hoc test was used (significance level *p* < 0.05). The correlation between the content of amino acids was expressed by Pearson’s correlation coefficient. The values of the Pearson’s correlation coefficients were selected and applied based on the values provided by Šimoliūnienė R. et al. [67].

## 5. Conclusions

This study highlights the potential of Lithuanian apple cultivars as valuable sources of free amino acids, including essential amino acids important for human nutrition. The observed variability in amino acid profiles between different apple parts suggests the importance of considering tissue-specific composition in nutritional evaluations and food product development. These findings open opportunities for the selection and breeding of apple cultivars with enhanced amino acid profiles, contributing to the development of functional foods and healthier diets.

Future research should focus on multi-seasonal and multi-regional studies to validate the current results under different environmental conditions. Additionally, exploring the relationship between amino acid content and other phytochemicals, such as phenolic compounds, may further reveal the nutritional and functional potential of apples. Such insights could support both agricultural innovation and public health strategies.

## Data Availability

Data are contained within the article.
